# Simultaneous EEG-fMRI during a neurofeedback task, a brain imaging dataset for multimodal data integration

**DOI:** 10.1038/s41597-020-0498-3

**Published:** 2020-06-10

**Authors:** Giulia Lioi, Claire Cury, Lorraine Perronnet, Marsel Mano, Elise Bannier, Anatole Lécuyer, Christian Barillot

**Affiliations:** 1 Univ Rennes, Inria, CNRS, Inserm, IRISA UMR 6074, F-3500 Rennes, France; 2 CHU Rennes, Service de Radiologie, Rennes, France

**Keywords:** Brain imaging, Biomedical engineering, Neuroscience

## Abstract

Combining EEG and fMRI allows for integration of fine spatial and accurate temporal resolution yet presents numerous challenges, noticeably if performed in real-time to implement a Neurofeedback (NF) loop. Here we describe a multimodal dataset of EEG and fMRI acquired simultaneously during a motor imagery NF task, supplemented with MRI structural data. The study involved 30 healthy volunteers undergoing five training sessions. We showed the potential and merit of simultaneous EEG-fMRI NF in previous work. Here we illustrate the type of information that can be extracted from this dataset and show its potential use. This represents one of the first simultaneous recording of EEG and fMRI for NF and here we present the first open access bi-modal NF dataset integrating EEG and fMRI. We believe that it will be a valuable tool to (1) advance and test methodologies for multi-modal data integration, (2) improve the quality of NF provided, (3) improve methodologies for de-noising EEG acquired under MRI and (4) investigate the neuromarkers of motor-imagery using multi-modal information.

## Abstract (summary)

**Table:** 

Measurement(s)	brain measurement • motor behavior
Technology Type(s)	functional magnetic resonance imaging • electroencephalography (EEG)
Factor Type(s)	task • subject
Sample Characteristic - Organism	Homo sapiens

Machine-accessible metadata file describing the reported data: 10.6084/m9.figshare.12251765

## Background & Summary

Neurofeedback (NF) consists in providing real-time information to a subject about his own brain activity in order to train self-regulation of specific brain areas and is a promising brain rehabilitation technique for psychiatric disorders^1,2^, stroke^3–5^ and other neurological pathologies^6^. NF approaches are usually based on real-time measures of brain activity using a single imaging technique, with the majority of applications relying on EEG and some recent ones employing functional imaging (e.g. functional MRI)^7,8^.

Recent studies^2,9^ have shown the potential of combining different imaging techniques to achieve a more specific self-regulation, the combination of two complementary modalities such as fMRI and EEG being particularly promising. fMRI offers fine spatial resolution (~mm)^10^ but has slow dynamics (~s). On the other hand, EEG provides an excellent temporal resolution (~ms), its spatial resolution being however limited (~cm) by cortical currents volume conduction through the head tissues^11^. These imaging modalities are highly complementary and their integration is promising in applications requiring high temporal and spatial resolution such as NF training of specific brain areas.

More generally, the joint analysis of different imaging modalities can shed light on the complex link between anatomical, functional and electrophysiological properties of the brain^12^. Integration of EEG and fMRI allows for an ‘’augmented” analysis of the neuronal dynamics, as the single modalities provide a partial estimation of the underlying neural activity. Joint EEG-fMRI analyses fall in two categories: asymmetrical and symmetrical^13^. In the first, information extracted from one methodology are integrated or drive the analysis of the second while in symmetrical approaches (data fusion^13^) a joint generative model is used. These approaches have been poorly explored, complexity of neurovascular coupling being their main limitation.

Collecting EEG in the ‘harsh’ environment of MR scanners entails a series of technical challenges, primarily devoted to the reduction of the strong EEG artefacts generated by MR gradient currents. The degradation of EEG probably represents the main issue of simultaneous EEG-fMRI recordings and several de-noising solutions have been developed^14,15^. However, so far, the corresponding datasets have rarely been released for use by other groups. Moreover, for the implementation of NF, simultaneous EEG-fMRI acquisition and processing need to be executed in real-time, adding complexity and challenge to hardware and software solutions.

**Fig. 1:**
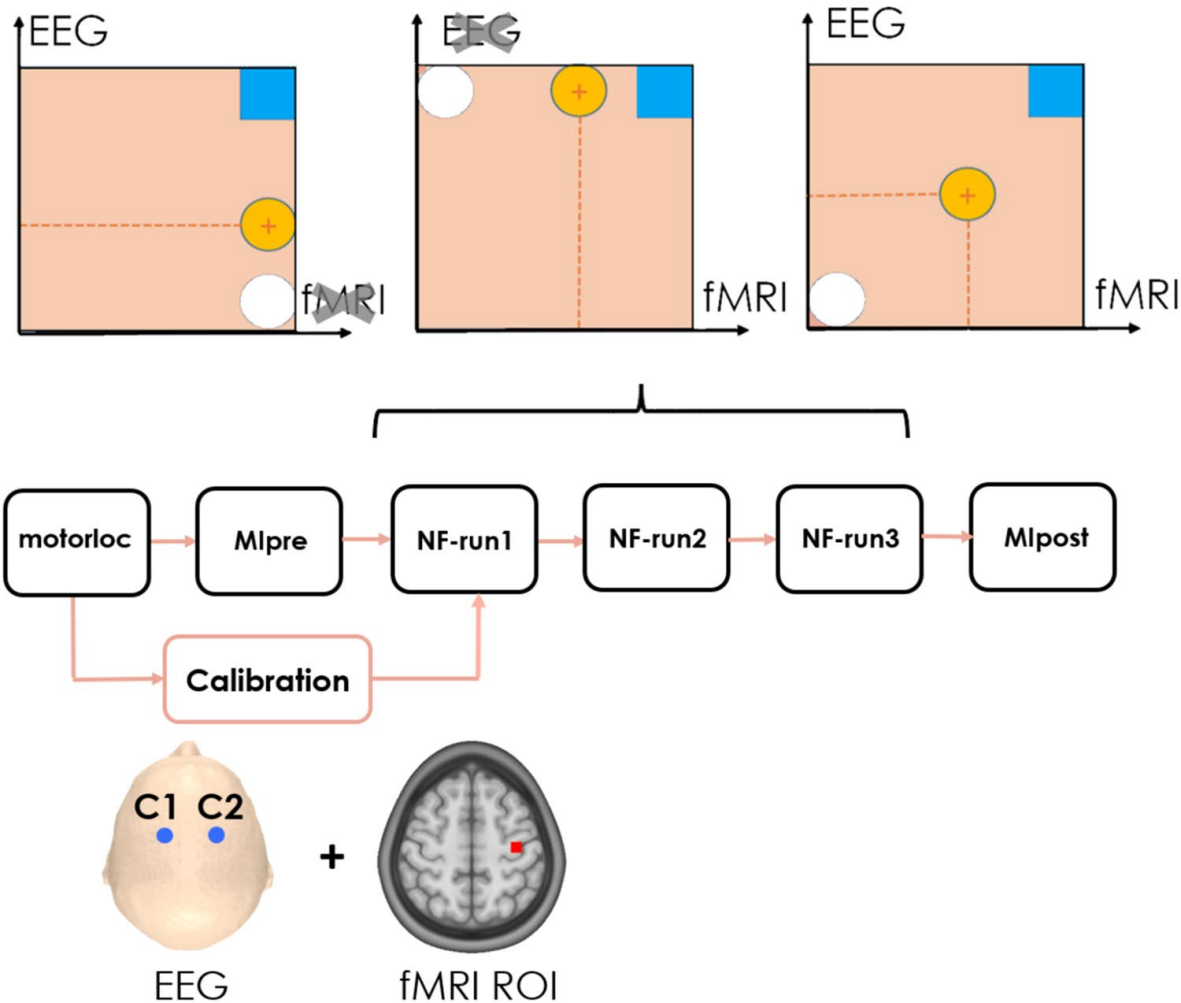
Experimental Protocol XP1.

**Fig. 2:**
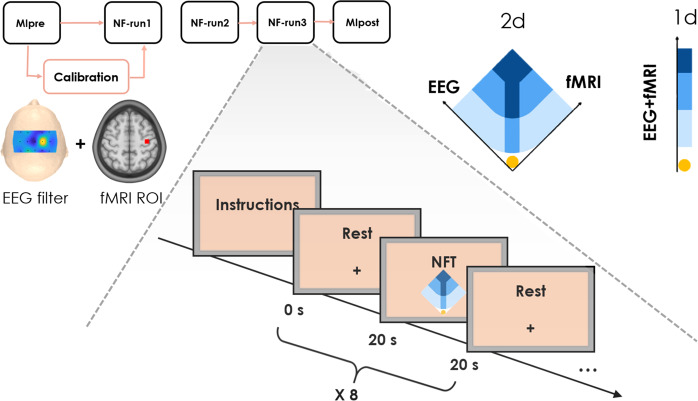
Experimental protocol XP2.

To the best of our knowledge, simultaneous EEG-fMRI in a NF loop was implemented only by another research group to train emotional self-regulation^2^: the dataset we share and describe here therefore corresponds to the first implementation of bimodal NF for a motor imagery task. It consists of 64-channels EEG (extended 10–20 system) and fMRI datasets simultaneously acquired during a motor imagery NF task, complemented by structural MRI scans. Recordings were performed in two studies. The first (Fig. 1) involved 10 healthy subjects and aimed at assessing the feasibility and the potential of bimodal EEG-fMRI NF over unimodal (EEG or fMRI only) NF^9^. In the second study^16^ that included 20 volunteers, an original 2D metaphor allowing to separately regulate EEG and fMRI activity was proposed and its impact on NF performance compared to a more “classical” 1D gauge (Fig. 2). An analysis of NF scores and BOLD activations was performed and results suggested that 1D feedback is easier to control, while the fMRI activation is more specific when the 2D representation is used.

Going beyond these previous works, we have BIDs standardized and comprehensively documented the two datasets: in this manuscript we describe in detail the procedures employed to generate data and the architecture of the dataset, we perform additional validation analysis, we give examples of the type of information that can be extracted and show various prospective uses. This dataset has potential to shed light on the coupling model underlying the EEG and fMRI signals, to advance methodologies for multimodal data integration and fusion techniques and test EEG MRI de-noising algorithms. Since data were acquired during the execution of a simple task for whom neural pathways and activations are well known (motor imagery), this dataset constitutes a simple model to develop and validate methods of data integration at various scales (activation maps, connectome).

This dataset is also of great interest for the NF research field, as it gives new perspectives to improve the estimation of NF scores, or the extraction of features of interest. As dataset derivatives, we computed EEG and fMRI NF scores for each training session. These can be used in a model to learn fMRI informed EEG in order to improve the quality of EEG only NF^17^. The development of such approaches has potential to increase the number and quality of NF training sessions and therefore advance clinical applications.

## Methods

### Participants

All experiments were performed according to the Helsinki declaration and under approval by the Institutional Review Board. Thirty right-handed NF-naïve healthy subjects were involved, of which 12 were female (mean age 33.7 ± 9.9 years old). All participants signed an informed consent after having been informed about the experimental procedure. Only 27 of them signed the consent to publish their anonymized data, and were therefore included in the public datasets^18,19^.

### Data acquisition

**Fig. 3:**
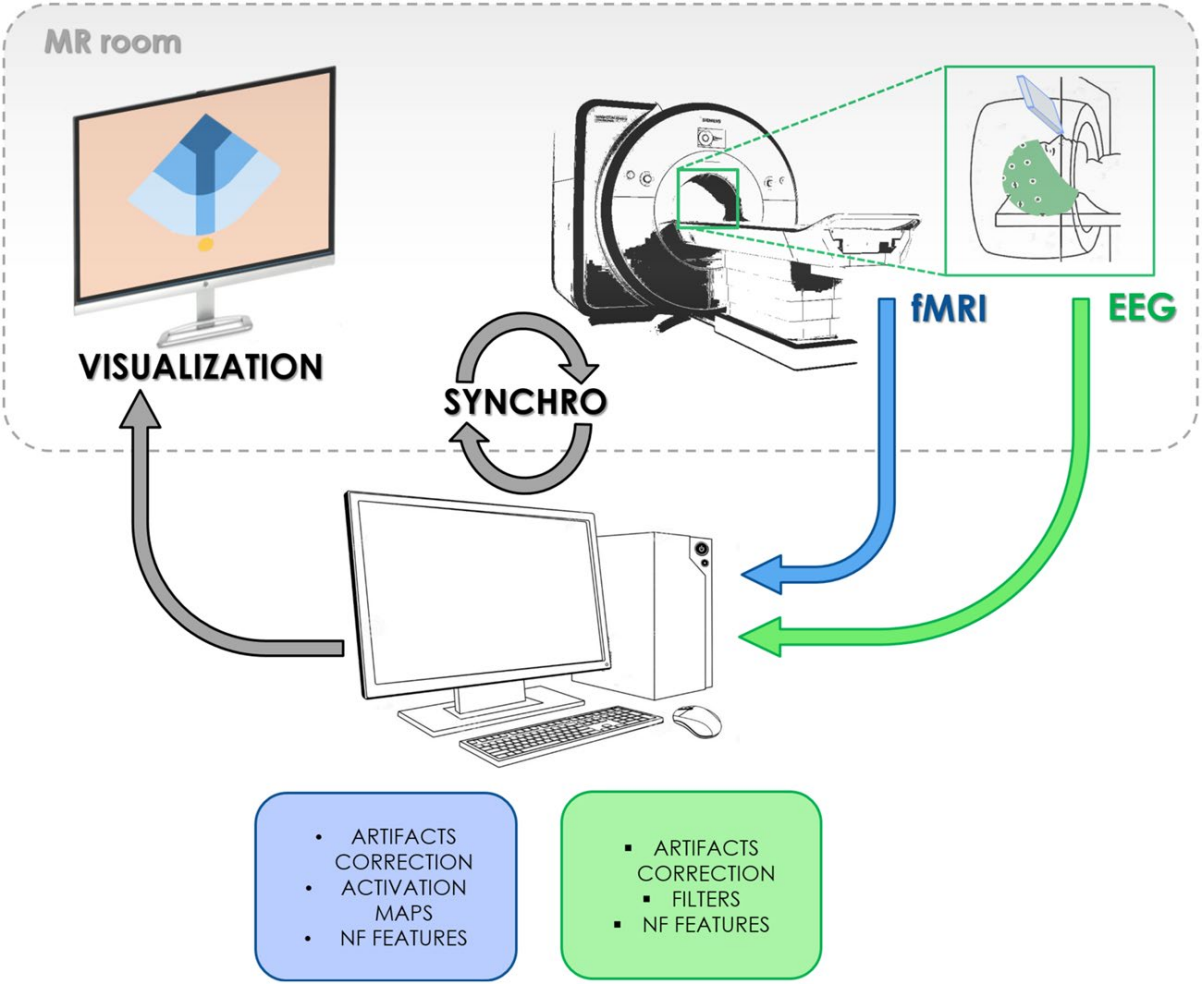
Schematic visualisation of the bimodal EEG-fMRI NF platform.

The multimodal imaging NF platform used to acquire simultaneously EEG and fMRI data and to provide real-time NF has been described in^20^ and a schematic is shown in Fig. 3. It guarantees the acquisition, processing, NF features computation and visualization in real-time of the two data streams (EEG and fMRI). A system of callbacks ensures full synchronization of the acquisition, stimuli and NF presentation processes. The platform includes a MR compatible, 64-channel extended international 10–20 EEG system, with FCz as the reference electrode and AFz as the ground electrode (Brain Products GmbH, Gilching, Germany) and a 3T Verio MRI running VB17 (Siemens Healthineers, Erlangen, Germany), equipped with a 12-channel receiver head coil.

In line with the EEG vendor guidelines, a great care was put in the installation of the 64-channel EEG cap and reduction of electrode impedances. Electrodes impedances values estimated at the beginning of each run can be found in the header of the respective EEG file, as explained in the Data Records session. The subjects was then installed in the MR bore and electrodes impedances verified again after installation. Foam pads were placed around the subject’s head in the 12-channel head coil to minimize head motion. For the whole duration of the experiment the subjects were asked to lie still in the MR bore. A LCD screen (NordicNeurolab Solutions, Bergen, Norway) was placed at the back of the MR scanner, a rear-facing mirror fixed on the top of the head coil allowed the subjects to view the screen.

For both studies, EEG recordings were sampled at 5 kHz with the reference electrode in FCz and ground in AFz. A 1 mm isotropic 3D T1 MPRAGE structural scan was acquired. fMRI acquisition was performed by means of echo-planar imaging (EPI) whose settings changed from the first to the second study, as the repetition time (TR) could be further decreased thus improving the BOLD NF updating rate. Details about EEG and MRI data can be found in the Data Records session.

### Experimental Paradigm

#### XP1

The experimental paradigm of the first study has been described in previous work^9^ and is illustrated in Fig. 1. Subjects were briefed about the task and familiarized with the NF metaphor (a ball moving in one or two dimensions depending on their brain activity). They were instructed to perform a kinaesthetic motor imagery (MI) of the right hand and to find their own strategy to control and bring the ball to the target. Specifically, they were informed that the NF would be a measure of laterality and that they had to maximize the activity only over the right hand motor region, therefore avoiding to image movements involving the two hands. Instructions were iterated verbally and on the screen at the beginning of NF run, together with the information about the direction (vertical, horizontal or both) along which the ball could be moved (Fig. 1a). Participants were asked to hold still inside the MR bore and video monitoring allowed checking for movements of the subjects.

The experiment included six runs of a block-design alternating rest (20 s) and task (20 s). Beforehand a motor localizer (motorloc) was performed where the subject was instructed to clench his right-hand every second during task, for a total duration of 5 min and 20 s (8 blocks, starting with rest). A preliminary MI (MIpre) without feedback was then executed for 3 min and 20 s (5 blocks). Three NF blocks followed where the subject performed EEG-NF only (eegNF), fMRI-NF (fmriNF) and EEG-fMRI-NF (eegfmriNF) in random order across subjects. Each of the NF training run consisted of 10 blocks (6 min and 40 s). During NF runs the screen displayed a white ball moving in the vertical (condition eegNF, EEG laterality depicted the ball ordinate), or horizontal (condition fmriNF, BOLD laterality depicted the ball abscissa) or both dimensions (condition eegfmriNF) and a square representing the target. Finally, a transfer block of MI without NF (MIpost) was performed (3 min and 20 s), with the rationale of assessing the NF learning effect with respect to MIpre.

#### XP2

The following methods section is an expanded version of a related work from our group^16^ and is schematically shown in Fig. 2. As for the first study, the experimental procedure and the NF task were carefully explained to participants before and during the experiment, in the form of written and verbal instructions. Similar instructions for kinaesthetic MI were given and the two NF metaphors (bi-dimensional 2d and unidimensional 1d) described. For the first group (1d) a ball moving on a mono-dimensional gauge was shown, and participants instructed to bring the ball towards the target (top, darkest areas of the gauge) by imagining moving their left hand. In the second group (2d) subjects could regulate separately the contribution of EEG and fMRI activities on the left and right axes, respectively (Fig. 2).

Firstly, a Mipre run (without feedback) was performed (5 min 20 s). Data collected from this session was used to identify the region-of-interest (ROI) for fMRI processing and the optimal EEG filter (see next section). Three NF runs followed, each of 5 min and 20 s and spaced by a 1 min break. A MIpost block without feedback concluded the session.

### Real-Time Data processing and NF computation

During simultaneous EEG-fMRI acquisition, two main types of artefacts critically compromise the quality of EEG recordings: the rapid switching of the magnetic field gradients generate the so-called “gradient artefact”, while the heartbeat-induced movements of EEG electrodes generate the ballistocardiogram (BCG) artefact. Real-time correction of EEG artefacts was performed using the Brain Vision Recview software 2.1.2 (Brain Products GmbH, Gilching, Germany). An average artefact subtraction approach^14^ was used for gradient artefact correction, with four artefact templates. EEG data were then down-sampled to 200 Hz and low-pass filtered at 50 Hz (48 dB slope) for further processing. BCG artefact correction was then performed^15^ using a moving template matching method with the following parameters: pulse period 800 ms, correlation threshold 0.7 and amplitude ratio between the period examined and the pulse model ranging from 0.6 to 1.2. The moving pulse template was built averaging the 10 previous detected pulses. The corrected EEG data were subsequently sent to the NF control unit for feature extraction via a fieldtrip (https://github.com/fieldtrip/fieldtrip/) buffer solution.

Real-time fMRI pre-processing (slice-time and motion correction) was performed directly by the NF control unit using a custom Matlab script based on SPM 8 (FIL, Wellcome Trust Centre for Neuroimaging, UCL, London, UK). More details, according to the COBIDAS-inspired template^21^, are reported in Table 1.

**Table 1:** Real-time fMRI preprocessing steps from the COBIDAS-inspired template: https://osf.io/kjwhf/.

	Real-time fMRI pre-processing	
	XP1	XP2
Slice-time correction	YES	YES
	SPM + Matlab	SPM + Matlab
	Performed after motion correction	Performed after motion correction
Motion correction	YES	YES
	SPM + Matlab	SPM + Matlab
	Rigid registration	Rigid registration
	Interpolation type: spline	Interpolation type: spline
Function-structure co-registration	No	No
Distortion correction	No	No
Spatial smoothing	YES	YES
	SPM + Matlab	SPM + Matlab
	Smoothing size: 3 mm	Smoothing size: 3 mm
	Fixed kernel	Fixed kernel
Nuisance regression	No	No
Drift removal	No	No
Physiological noise removal	YES	YES
	SPM + Matlab	SPM + Matlab
	Differential region of interest: contralateral motor cortex percent signal change subtracted from the ipsilesional ROI percent signal change at each NF update	Differential region of interest: Average signal on a “deep slice” (slice #6) subtracted from the ROI percent signal change at each NF update
High frequency filtering	No	No
Volume censoring	No	No
Serial correlations	No	No
Temporal averaging	YES	YES
	SPM + Matlab	SPM + Matlab
	3 scans moving average window	3 scans moving average window
Intensity normalisation	No	No
Real-time data quality control	YES	YES
	SPM + Matlab	SPM + Matlab
	Real-time display of head motion parameters (3 rotations, 3 axes) to researcher	Real-time display of head motion parameters (3 rotations, 3 axes) to researcher
Offline data quality control	YES	YES
	SPM + Matlab + SPMup	SPM + Matlab + SPMup
	Framewise displacement calculation and correlation analysis (NF scores, head motions)	Framewise displacement calculation and correlation analysis (NF scores, head motions)

#### XP1

Data collected during the motorloc session were used to identify a ROI for fMRI NF computation. fMRI scans were pre-processed for slice-time correction, spatial realignment and smoothing with SPM8 and activation maps were estimated with a GLM approach. A ROI over the left M1 was defined as the 9 × 9 × 3 voxels surrounding the maximum of activation. The right M1 ROI was defined as the symmetrical ROI across the mid sagittal plane. The activations from these two ROIs were included in the computation of the laterality NF feature and fed back to the subject during NF training. The EEG NF feature, on the other hand, was computed considering the EEG power in the (8–12) Hz band in the C1 and C2 electrodes (see the section NF features computation).

After pre-processing, EEG feature was computed according to Eq. (1):

1 $${{\bf{NF}}}_{{\bf{eeg}}}({\bf{t}})=[{{\bf{BP}}}_{{\bf{left}}}\left({\bf{t}}\right)-{{\bf{BP}}}_{{\bf{right}}}({\bf{t}})]/[{{\bf{BP}}}_{{\bf{left}}}\left({\bf{t}}\right)+{{\bf{BP}}}_{{\bf{right}}}({\bf{t}})]$$

where ${{\bf{BP}}}_{{\bf{left}}}\left({\bf{t}}\right)$ (respectively ${{\bf{BP}}}_{{\bf{right}}}({\bf{t}})$) is the band power (estimated by applying a 2 s Hamming window and using a periodogram) in the 8–12 Hz range at C1 (respectively C2) during the NF task normalized by the power during rest. In other words, ${{\bf{NF}}}_{{\bf{eeg}}}\left({\bf{t}}\right)$ is a measure of the MI desynchronization laterality. The EEG laterality index was then smoothed by averaging over the last 6 values, normalized and eventually translated as the ordinate of the NF ball and updated every 250 ms.

The fMRI laterality index was calculated on the corrected scans according to Eq. (2):

2 $${{\bf{NF}}}_{{\bf{fmri}}}({\bf{v}})=\frac{{{\bf{B}}}_{{\bf{left}}}\left({\bf{v}}\right)}{{{\bf{B}}}_{{\bf{left}}}\left({\bf{rest}}\right)}-\frac{{{\bf{B}}}_{{\bf{right}}}\left({\bf{v}}\right)}{{{\bf{B}}}_{{\bf{right}}}\left({\bf{rest}}\right)}$$

Here ${{\bf{B}}}_{{\bf{left}}}\left({\bf{v}}\right)$ is the average of the BOLD signal in the left motor ROI identified during calibration at volume v. ${{\bf{B}}}_{{\bf{left}}}\left({\bf{v}}\right)$ is then normalized by the average signal in the same ROI over the last 6 volumes of the resting block ${{\bf{B}}}_{{\bf{left}}}\left({\bf{rest}}\right)$. The fMRI laterality index was then smoothed by averaging it over the last three volumes and translated in the ordinate of the ball in the NF animation. The fMRI NF dimension was updated every 2 s (TR).

During rest blocks, a fixation cross was displayed and participants were asked to rest. During task, the screen displayed a cue (“move right”/“imagine right”) as well as the NF metaphor consisting in a ball moving along the horizontal (fmriNF) axis, along the vertical (eegNF) axes or along both axes (Fig. 1).

#### XP2

For the second study, a more sophisticated calibration procedure was performed:data collected during the MIpre session were used to identify an optimal spatial filter for EEG and a ROI based on the maximum of activation in the target motor area. As described in detail in^16^, EEG data were pre-processed in Analyzer (Brain Products GmbH, Gilching, Germany) and BCG artefacts were corrected with a semi-automated procedure. A Common Spatial Pattern (CSP) filter maximizing the difference in EEG power in the target band (8–30 Hz) between rest and MI blocks was estimated from a selection of motor channels. fMRI scans were pre-processed for slice-time correction, spatial realignment and smoothing with SPM8 and activation maps were estimated with a GLM approach. The selected ROI was defined as the ROI (9 × 9 × 3 voxels) around the peak of activation in the target motor area (i.e. left motor cortex).

In XP2, EEG NF score was a measure of event-related desynchronization (ERD)^22^:

3 $${{\bf{NF}}}_{{\bf{eeg}}}({\bf{t}})=[{{\bf{BP}}}_{{\bf{left}}}\left({\bf{rest}}\right)-{{\bf{BP}}}_{{\bf{left}}}({\bf{t}})]/{{\bf{BP}}}_{{\bf{left}}}\left({\bf{rest}}\right)$$

where ${{\bf{BP}}}_{{\bf{left}}}({\bf{t}})$ is the power in the 8–30 Hz frequency band and the ${{\bf{BP}}}_{{\bf{left}}}\left({\bf{rest}}\right)$ is the average power over the resting block preceeding the NF training. ${{\bf{NF}}}_{{\bf{eeg}}}\left({\bf{t}}\right)$ is a measure of the desynchronization occurring during MI with respect to the baseline at rest. EEG NF scores were then smoothed over the last 4 values, normalized and converted in the visual feedback (ball movement) every 250 ms.

The fMRI feedback was calculated according to the following formula:

4 $${{\bf{NF}}}_{{\bf{fmri}}}({\bf{v}})=\frac{{{\bf{B}}}_{{\bf{ROI}}}\left({\bf{v}}\right)}{{{\bf{B}}}_{{\bf{ROI}}}\left({\bf{rest}}\right)}-\frac{{{\bf{B}}}_{{\bf{BG}}}\left({\bf{v}}\right)}{{{\bf{B}}}_{{\bf{BG}}}\left({\bf{rest}}\right)}$$

${{\bf{B}}}_{{\bf{ROI}}}\left({\bf{v}}\right)$ is the fMRI signal in the motor ROI selected in the calibration step at volume v and is divided by the corresponding signal averaged across the last 6 s of the previous rest block (to take into account the hemodynamic delay). ${{\bf{B}}}_{{\bf{BG}}}\left({\bf{v}}\right)$ is the BOLD signal in a background lower slice, used here in order to normalize by global BOLD signal changes as recommended in^23^. The fMRI NF index was averaged over the last three volumes and translated in a movement of the ball in the NF animation every second.

During NF runs the screen displayed instructions and the feedback which consisted of a ball moving in a two-dimensional plot for the 2d group or in a one-dimensional gauge for the 1d group (Fig. 1b).

A CRED-nf checklist^24^ summarizing experimental design together with a series of comments to specific items of the list is available respectively in Supplementary File 1 (CRED-nf) and Supplementary File 2 (CRED-nf comments).

## Data Records

The anonymized datasets are publicly available on the OpenNeuro repository in BIDS format^25^. Even if the two datasets are very similar and, depending on the scientific purpose, can be jointly analysed, they were split in XP1^18^ and XP2^19^ for the sake of clarity.

### XP1

**Fig. 4:**
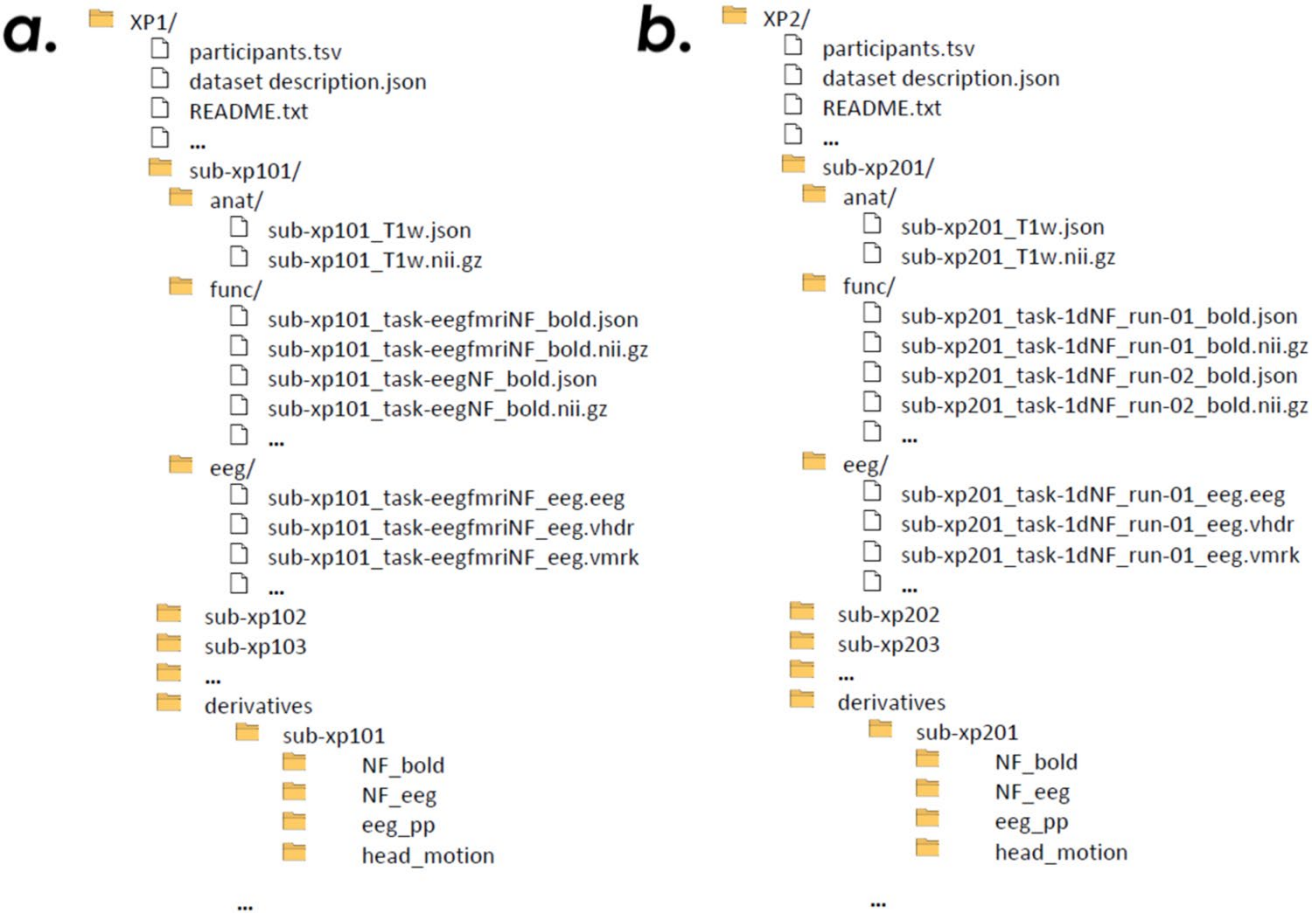
Folders architecture of XP1 (**a**) and XP2 (**b**).

The resource contains data from 10 subjects (18 GB), as summarised in Table 2. The folder layout follows (both for MRI and EEG data) the BIDS specification folder hierarchy (Fig. 4a). https://doi.org/10.18112/openneuro.ds002336.v2.0.0.

**Table 2:** Participant demographic for XP1. Participants ID age and sex is indicated, together with the NF tasks listed in the order they performed them.

participant_id	age	sex	feedback_task
sub-xp101	25	M	fmri-nf, eeg-nf, eegfmri-nf
sub-xp102	27	M	eeg-nf, eegfmri-nf, fmri-nf
sub-xp103	25	M	fmri-nf, eeg-nf, eegfmri-nf
sub-xp104	31	M	fmri-nf, eegfmri-nf, eeg-nf
sub-xp105	39	M	eegfmri-nf, fmri-nf, eeg-nf
sub-xp106	36	F	eegfmri-nf, eeg-nf, fmri-nf
sub-xp107	19	M	eeg-nf, fmri-nf, eegfmri-nf
sub-xp108	29	M	eeg-nf, fmri-nf, eegfmri-nf
sub-xp109	27	F	fmri-nf, eegfmri-nf, eeg-nf
sub-xp110	26	M	eegfmri-nf, fmri-nf, eeg-nf

### EEG Data

#### Raw EEG

Raw EEG is sampled at 5 kHz with FCz as the reference electrode and AFz as the ground electrode, and a resolution of 0.5 µV. Following the BIDS arborescence, raw EEG data for each task and subject can be found in

XP1/sub-xp1*/eeg

in Brain Vision Recorder format (File Version 1.0). Each raw EEG recording includes three files: the data file (*.eeg), the header file (*.vhdr) and the marker file (*.vmrk). The header file contains information about acquisition parameters and amplifier setup. For each electrode, the impedance at the beginning of the recording is also specified, giving an indication of the quality of recording settings. For all subjects, channel 32 is the ECG channel. The 63 other channels are EEG channels. The marker file contains the list of markers assigned to the EEG recordings and their properties (marker type, marker ID and position in data points). Three type of markers are relevant for the EEG processing:

R128 (Response): is the fMRI volume marker to correct for the gradient artefact

S 99 (Stimulus): is the protocol marker indicating the start of the Rest block

S 2 (Stimulus): is the protocol marker indicating the start of the Task (i.e. Motor Execution Motor Imagery or Neurofeedback). For technical reasons and for a few runs, the first S99 marker was not correctly recorded. However, it can be easily identified 20 s before the first S 2.

#### Preprocessed EEG

Following the BIDS structure, processed EEG data for each subject and task can be found in the pre-processed data folder:

XP1/derivatives/sub-xp1*/eeg_pp/*eeg_pp.*

in Brain Analyzer format. Each processed EEG recording includes three files: the data file (*.dat), the header file (*.vhdr) and the marker file (*.vmrk), containing information similar to those described for raw data. In the header file of preprocessed data, channels locations are also specified. In the marker file the location in data points of the identified heart pulse (R marker) are specified as well.

EEG data were pre-processed using BrainVision Analyzer II Software, with the following steps:

- Automatic gradient artefact correction using the artefact template subtraction method (sliding average calculation with 21 intervals for sliding average and all channels enabled for correction).
- Low Pass FIR Filter 50 Hz.
- Downsampling with factor 25 (200 Hz)
- BCG artefact correction using a semiautomatic procedure (Template searched between 40 s and 240 s in with the following parameters: Coherence Trigger = 0.5, Minimal Amplitude = 0.5, Maximal Amplitude = 1.3. The identified pulses were marked with R).
- Segmentation relative to the first block marker (S 99) for all the length of the training protocol (last S 2 + 20 s).

Both raw and preprocessed EEG data can be analyzed with common EEG software solutions, as the BrainVision file format is supported by many open source packages (e.g. FieldTrip, EEGLAB, SPM, MNE-Python).

#### EEG NF scores

Neurofeedback scores can be found in the.mat structures in

XP1/derivatives/sub-xp1*/NF_eeg/d_sub*NFeeg_scores.mat

NF_eeg structures are composed of the following subfields:

NF_eeg:

→ .nf_laterality (NF score computed as for real-time calculation - Eq. 1)

→ .filteegpow_left (Bandpower of the filtered eeg signal in C1)

→ .filteegpow_right (Bandpower of the filtered eeg signal in C2)

→ .nf (vector of NF scores -4 per second- computed as in Eq. 3) for comparison with XP2

→ .smoothed

→ .eegdata (64 X 200 X 400 matrix, with the pre-processed EEG signals according to the steps described above)

→ .method

Where the subfield method contains information about the laplacian filtered used and the frequency band of interest.

### MRI Data

All DICOM files were converted to Nifti-1 and then in BIDS format (version 2.1.4) using dcm2niix (https://github.com/rordenlab/dcm2niix, version v1.0.20190720 GVV7.4.0)

#### Raw functional MRI

Following the BIDS architecture (Fig. 4a), the functional data and relative metadata are found for each subject in the following directory

XP1/sub-xp1*/func

fMRI acquisitions were performed using echo-planar imaging (EPI) and covering the entire brain with the following parameters: TR = 2 s, TE = 23 ms, Resolution 2 × 2 × 4 mm^3^, FOV = 210 × 210 mm^2^, Number of 4-mm slices: 32, no slice gap.

As specified in the relative task event files in XP1\*events.tsv files onset, the scanner began the EPI pulse sequence two seconds prior to the start of the protocol (first rest block), so the first TR should be discarded. In task events files for the different tasks, each column represents:

- ‘onset’: onset time (sec) of an event
- ‘duration’: duration (sec) of the event
- ‘trial_type’: trial (block) type: rest or task (Rest, Task-ME, Task-MI, Task-NF)
- ‘stim_file’: image presented in a stimulus block: during Rest, Motor Imagery (Task-MI) or Motor execution (Task-ME) instructions were shown. On the other hand, during Neurofeedback blocks (Task-NF) the image presented was a ball moving in a square that the subject could control self-regulating his EEG and/or fMRI brain activity.

#### fMRI NF Scores

For each subject and NF session, a Matlab structure with BOLD-NF features can be found in

XP1/derivatives/sub-xp1*/NF_bold/

In view of BOLD-NF scores computation, fMRI data were preprocessed using SPM8 according to the following steps: slice-time correction, spatial realignment and coregistration with the anatomical scan, spatial smoothing with a 6-mm isotropic Gaussian kernel and normalization to the Montreal Neurological Institute (MNI) template.

For each session, a first level GLM analysis was then performed. The resulting activation maps (voxel-wise Family-Wise error corrected at p < 0.05) were used to define two ROIs (9 × 9 × 3 voxels) around the maximum of activation in the left and right motor cortex. The BOLD-NF scores (fMRI laterality index) were calculated as the difference between percentage signal change in the left and right motor ROIs as for the online NF calculation. A smoothed and normalized version of the NF scores over the precedent three volumes was also computed. To allow for comparison and aggregation of the two datasets XP1 and XP2, NF scores were also computed considering the left motor cortex and a background as for online NF calculation in XP2.

In the NF_bold folder, the Matlab files sub-xp1*_task-*_NFbold_scores.mat have the following structure:

NF_bold

→ .nf_laterality (calculated as for online NF calculation)

→ .smoothnf_laterality

→ .normnf_laterality

→ .nf (calculated as for online NF calculation in XP2)

→ .roimean_left (averaged fMRI signal in the left motor ROI)

→ .roimean_right (averaged fMRI signal in the right motor ROI)

→ .bgmean (averaged fMRI signal in the background slice)

→ .method

Where the subfield “.method” contains information about the ROI size (.roisize), the background mask (.bgmask) and ROI masks (.roimask_left,.roimask_right). More details about signal processing and NF calculation can be found in^9^.

#### Anatomical MRI data

Following the BIDS standard, the functional data and relative metadata are found for each subject in the following directory

XP1/sub-xp1*/anat

As a structural reference for the fMRI analysis, a high resolution 3D T1 MPRAGE sequence was acquired with the following parameters: TR = 1900 ms, TE = 2.26 ms, TI = 900 ms, Resolution 1 × 1 × 1 mm^3^, Parallel Imaging GRAPPA with a Factor of 2, FOV = 256 × 256 mm^2^, Number of slabs: 176.

Defacing of 3D T1 MR-images was performed using pydeface (https://github.com/poldracklab/pydeface).

### XP2

**Fig. 5:**
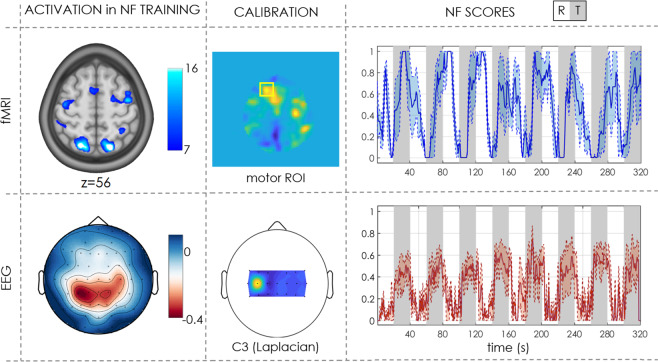
Example of fMRI and EEG activation and NF scores for one subject (XP2). The first column represents the activation (BOLD contrast for fMRI and ERD scalp distribution for EEG) during NF training. The second column show calibration features (ROI in the contralateral motor cortex for fMRI NF computation and Laplacian around the C3 electrode for EEG NF respectively). In the last column average (mean + std error) NF scores across 3 NF sessions are shown (rest blocks in white and NF task blocks in grey).

The resource contains data from 17 subjects (~26 GB), as summarized in Table 3. Some subjects who participated to the study refused to make their data publicly available and were not included in the repository. The folder layout follows, both for MRI and EEG data, the BIDS format (Fig. 5). 10.18112/openneuro.ds002338.v2.0.0.

**Table 3:** Participant demographic for XP2. Participants ID, age and sex is indicated, together with the NF tasks listed in the order they performed them.

participant_id	age	sex	feedback_task
sub-xp201	41	F	1d eegfmri-nf (3 runs)
sub-xp202	39	M	1d eegfmri-nf (3 runs)
sub-xp203	32	M	1d eegfmri-nf (3 runs)
sub-xp204	34	F	2d eegfmri-nf (3 runs)
sub-xp205	28	F	2d eegfmri-nf (3 runs)
sub-xp206	31	M	1d eegfmri-nf (3 runs)
sub-xp207	39	M	2d eegfmri-nf (3 runs)
sub-xp210	26	F	2d eegfmri-nf (3 runs)
sub-xp211	50	M	1d eegfmri-nf (3 runs)
sub-xp213	31	M	2d eegfmri-nf (3 runs)
sub-xp216	31	M	2d eegfmri-nf (3 runs)
sub-xp217	36	F	2d eegfmri-nf (3 runs)
sub-xp218	46	F	1d eegfmri-nf (3 runs)
sub-xp219	26	F	1d eegfmri-nf (3 runs)
sub-xp220	66	M	1d eegfmri-nf (3 runs)
sub-xp221	42	M	2d eegfmri-nf (3 runs)
sub-xp222	32	F	1d eegfmri-nf (3 runs)

### EEG Data

EEG data were recorded, processed and saved as for XP1.

#### EEG NF scores

Neurofeedback scores can be found in the .mat structures in

XP2/derivatives/sub-xp2*/NF_eeg/d_sub*NFeeg_scores.mat

NF_eeg structures are composed of the following subfields:

→ .filteegpow_left (Bandpower of the laplacian filtered eeg signal in C3)

→ .nf (vector of NF scores -4 per second- computed as in Eq. 3)

→ .smoothnf

→ .eegdata (64 × 200 × 400 matrix, with the pre-processed EEG signals according to the steps

described above)

→ .method

Where the subfield method contains information about the laplacian filtered used and the frequency band of interest.

### MRI Data

All DICOM files were converted to Nifti-1 and then in BIDS format (version 2.1.4) using the software dcm2niix (version v1.0.20190720 GVV7.4.0).

#### Functional MRI

fMRI acquisition in XP2 slightly changed with respect to XP1. We used EPI covering only the superior half of the brain to increase the fMRI temporal resolution with the following parameters: TR = 1 s, TE = 23 ms, Resolution 2 × 2 × 4 mm^3^, Number of 4-mm slices: 16, no slice gap. As for XP1 and as specified in the relative task event files in XP2/*events.tsv, the scanner began the EPI pulse sequence two seconds prior to the start of the protocol (first rest block), so the first two TRs should be discarded.

In task events files for the different tasks, each column represents:

- ‘onset’: onset time (sec) of an event
- ‘duration’: duration (sec) of the event
- ‘trial_type’: trial (block) type: rest or task (Rest, Task-MI, Task-NF)
- ‘stim_file’: image presented in a stimulus block.

During Rest or Motor Imagery (Task-MI) instructions were presented to the subject. During Neurofeedback blocks (Task-NF) the image presented was a ball moving in a square for the bidimensional NF (task-2dNF) or a ball moving along a gauge for the unidimensional NF (task-1dNF) that the subject could control self-regulating his EEG and fMRI brain activity.

#### fMRI NF Scores

For each subject and NF session, a Matlab structure with fMRI-NF features can be found in

XP2/derivatives/sub-xp2*/NF_bold/

In the original work, in view of fMRI-NF scores computation, fMRI data were preprocessed using AutoMRI, a home-made software based on spm8 automating the preprocessing according to the following steps: slice-time correction, spatial realignment and coregistration with the anatomical scan, spatial smoothing with a 6-mm isotropic Gaussian kernel and normalization to the Montreal Neurological Institute (MNI) template.

For each session, a first level GLM analysis was then performed. The resulting activation maps (voxel-wise Family-Wise error corrected at p < 0.05) were used to define two ROIs (9 × 9 × 3voxels) around the maximum of activation in the ipsilesional primary motor area (M1) and supplementary motor area (SMA) respectively.

The BOLD-NF scores were calculated as the difference between percentage signal change in the two ROIs (SMA and M1) and a large background region (slice 6 out of 16) whose activity is not correlated with the NF task. A smoothed version of the NF scores over the precedent three volumes was also computed.

The NF_bold structure is the following

NF_bold

→ .m1

        → .nf

       → .smoothnf

       → .roimean (averaged BOLD signal in the ROI)

       → .bgmean (averaged BOLD signal in the background slice)

       → .method

→ .sma

       → .nf

       → .smoothnf

       → .roimean (averaged BOLD signal in the ROI)

       → .bgmean (averaged BOLD signal in the background slice)

       → .method

Where the subfield method contains information about the ROI size (.roisize), the background mask (.bgmask) and ROI mask (.roimask). More details about signal processing and NF calculation can be found in^16^.

#### Anatomical MRI data

Anatomical scans were acquired and defaced as for XP1.

## Technical Validation

The bimodal NF platform architecture for simultaneous EEG-fMRI recording is described in^20^, together with details about the two-layer data synchronization and preprocessing implemented in order to guarantee real-time NF presentation. Results relative to XP1^18^ were published in^9^, a study where the added value of bimodal NF with respect to the single modalities (EEG and fMRI only NF) was shown. XP2^19^ experiment was intended to characterize a more subtle aspect: the impact of visual representation (2D or 1D) of bimodal NF on the NF performances^16^. We also recently tested the feasibility of EEG-fMRI NF for stroke rehabilitation in a pilot study^5^.

An offline data quality control was performed to assess for the impact of movement artifacts on data quality and NF performances. Motion parameters were reported and used to perform a post hoc correlation analysis with fMRI and EEG NF scores calculated as in real-time. To this end and for each NF training session the Framewise Displacement (FD) was computed from the six realignment parameters using the spmup tool within the QAP Package (https://github.com/CPernet/spmup, http://preprocessed-connectomes-project.org/quality-assessment-protocol/index.html) as for Power *et al*.^26^. A correlation analysis between the FD time-series and the NF signals was then performed. A Matlab structure with the results of this analysis can be found in derivatives/sub-xp*/head_motion. It is composed of the the following fields

FD

→ .vector (FD time-series)

→ .outliers (FD S-outliers based on the median of pairwise distances)

→ .NFbold_ correlation

       → .ro (Pearson coefficient between fMRI NF scores and FD)

       → .p (p-values of significant correlation hypothesis testing)

→ .NFeeg_correlation

       → .ro (Pearson coefficient between eeg NF scores and FD)

       → .p (p-values of significant correlation hypothesis testing)

Globally, the module of the Pearson correlation between fMRI NF scores and FD was always lower than 0.3 (an arbitrary chosen threshold level indicating fairly low correlation) in XP1 and higher than 0.3 in 1.65% of the NF training sessions for XP2. Head motions artifacts correlated to a slightly larger extent with EEG NF scores, as correlation was found higher than 0.3 in 3.3% of cases for XP1 and 5% for XP2.

To illustrate the type of data and results we can extract from the datasets we show in Fig. 5 an example of fMRI and EEG activation and NF scores for one subject (XP2). BOLD activation maps typically show activation of primary and supplementary motor areas and visual areas during the NF task as compared to rest. Similarly, the average ERD (8–30 Hz) scalp distribution for EEG presents a desynchronization over the motor electrodes, in particular in the contralateral region. BOLD NF scores show quite high (and consistent across sessions) activation of the selected ROI during the NF task, while EEG NF scores are lower and exhibit larger variability across sessions (which is to be expected given the higher NF update rate for EEG and the residual artefacts that affect EEG quality). The quality of EEG acquisition for each subject and session can also be verified in the corresponding header file where the impedance value for each electrode at the beginning of the recording is indicated: in all recordings the impedance was kept below 20 kΩ and in most cases values are below 10 kΩ.

**Fig. 6:**
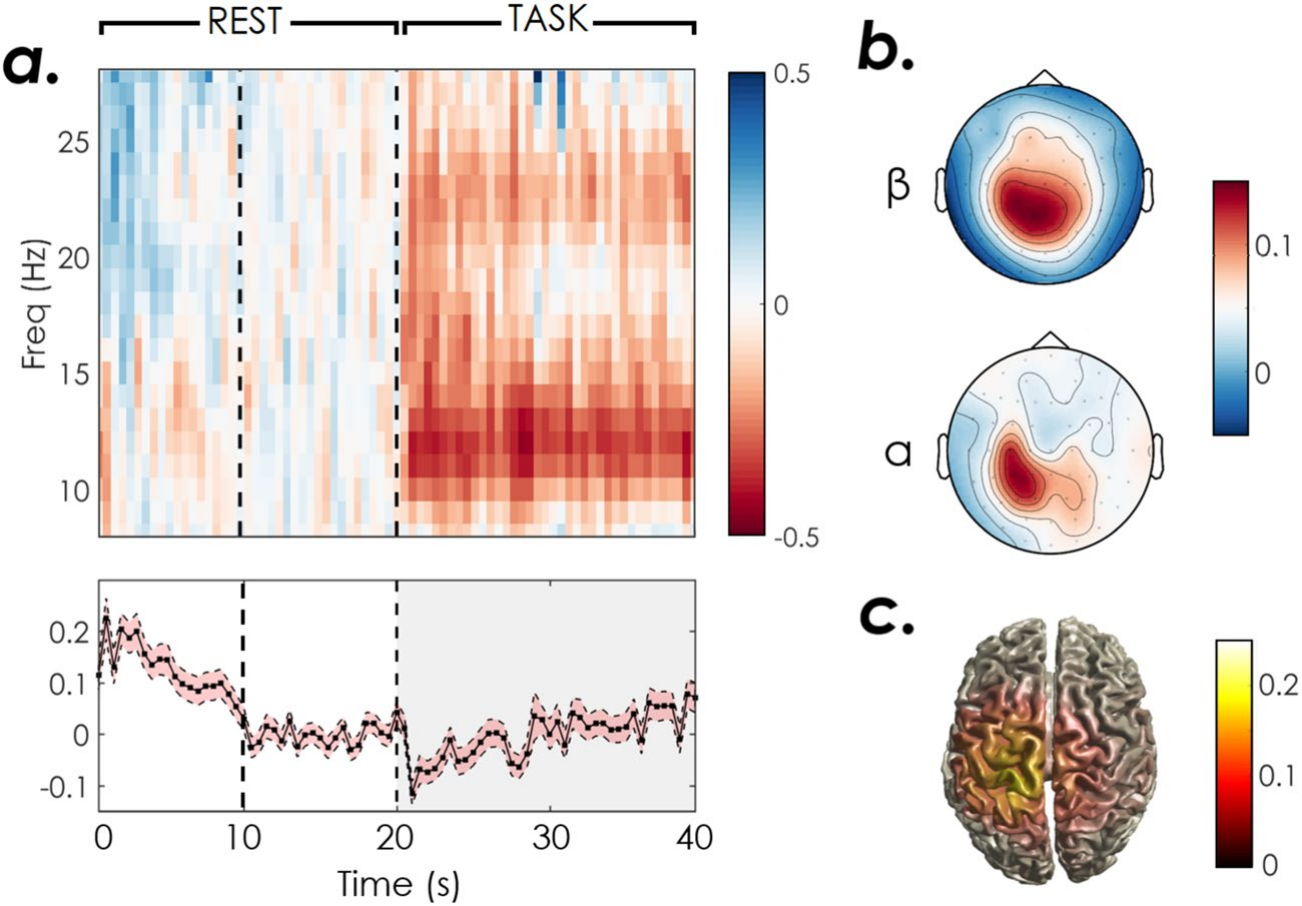
Average EEG ERD time-frequency patterns during NF training in XP2 (N = 18 subjects). (**a**) Time-Frequency maps showing ERD (red) and ERS (blue) in the contralateral motor electrode and the corresponding ERD time series averaged across all frequencies (mean + standard error across subjects). (**b**) ERD topographic maps in the alpha [8–12] Hz and beta [13–30] Hz frequency bands. (**c**) ERD cortical maps.

To validate the data quality a classic analysis of EEG and fMRI patterns was performed over the 20 participants of XP2. EEG power spectrum was first estimated using a multitaper Hanning approach in the 8–30 Hz frequency band. ERDs for each block and NF run were then computed. The baseline for ERD computation was computed in the last 10 s of rest (to exclude from the computation the event related synchronization occurring after motor imagery in the first seconds of the rest block, Fig. 6). We performed a k-means cluster analysis on individual ERD and ERS features and identified two outliers (xp211, xp212) showing, at least in one NF run, artefactual ERD. These were excluded from further analysis of EEG patterns. Average ERD scalp distributions in the alpha (8–12 Hz) and beta (13–30 Hz) frequency bands were investigated, as well as temporal and frequency patterns, as shown in Fig. 6. In line with results in literature, the average time-frequency map shows a desynchronization in the alpha band during the task block (and a less marked one in the beta band), together with a synchronization of the beta rhythm at the end of the motor task (i.e. in the first seconds of the rest block). Moreover, ERD scalp distributions indicate that EEG activity involves mainly contralateral motor electrodes and is more specifically localized (C3, CP3) in the alpha band. We also performed EEG source reconstruction using an eLoreta approach and the template head model implemented in fieldtrip. A boundary element method (BEM) volume conduction model based on the template MRI (semiautomatically aligned with EEG fiducials) was computed and used for inverse model estimation. Average source ERD results (Fig. 6c) indicate a desynchronization over the precentral end postcentral left gyri (corresponding to the sensory-motor cortex of the right upper limb).

fMRI analysis was performed using a processing pipeline based on Nipype, an open-source neuroimaging software that facilitates workflow reproducibility (https://github.com/nipy/nipype, version 1.2.0, Python 3.6). Preprocessing steps included slice timing and motion correction, detection of motion and intensity outliers, segmentation of anatomical image and coregistration, smoothing (fwhm = 6). A fist-level and second-level GLM analysis were also performed across the three NF runs (canonical HRF, voxel-based analysis, Family-Wise error (FWE) correction p = 0.05). Individual results were normalized to the Montreal Neurological Institute (MNI) template before performing a one-sample T-test group analysis. More details about MRI data processing can be found in the processing scripts available on github (https://github.com/glioi/BIDS_fMRI_analysis_nipype) together with schematics of the workflow steps.

**Fig. 7:**
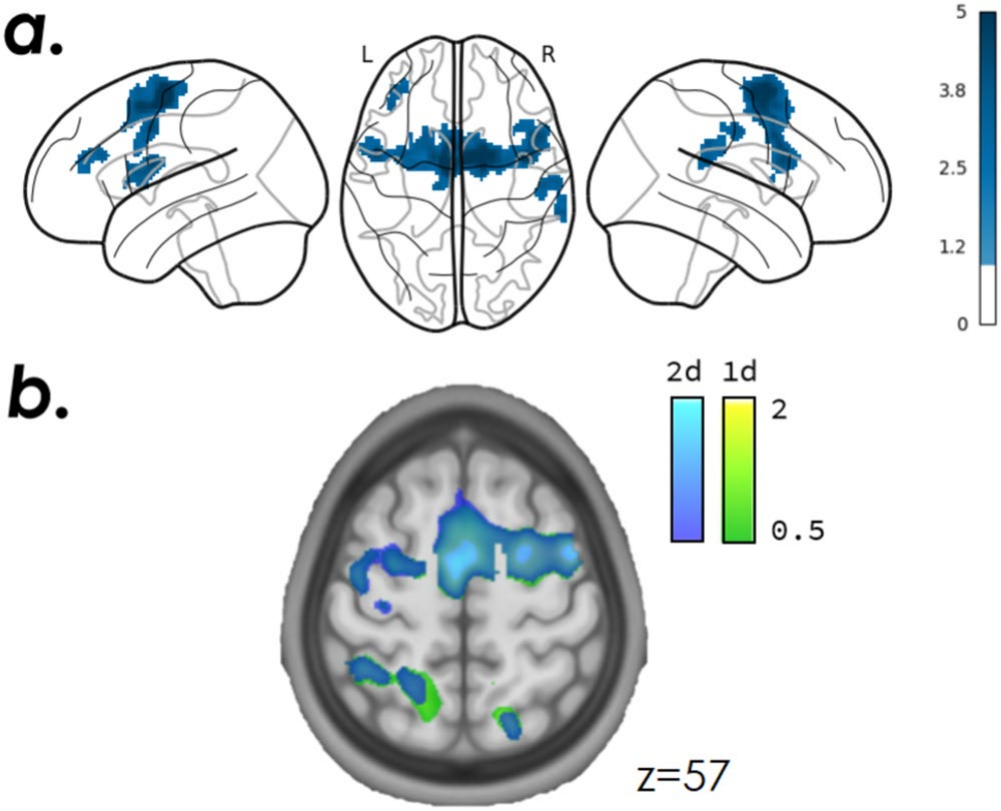
Group fMRI responses over three NF runs in XP2 (NF task > 0, p = 0.05 FWE corrected, voxel-based analysis). (**a**) Average activation maps (N = 20) thresholded at T-value = 1. (**b**) Contrast maps for the 2dNF group (blue) and 1dNF group (green).

The motion-related and intensity outliers were detected using the ArtefactDetection tool implemented in Nipype (motion threshold = 2, intensity z-score threshold = 2): an average of 12.1 ± 5.3 scans per NF training run (corresponding to 3.3% of artefactual scans per session) were detected and excluded from the following GLM analysis. In line with results in literature, average BOLD activation maps (Fig. 7) show significant activations in the premotor cortex and supplementary motor area (left and right), and in the left primary motor cortex during the motor NF task. These areas are involved in the planning and imagination of movement. The left posterior parietal cortex (PPC), that plays an important role in visuo-motor coordination, was also recruited during the motor imagery NF task, in line with influencing literature^6^ indicating that PPC is generally active when feedback is presented visually. Results are quite robust across the two groups (2dNF and 1dNF) that exhibit largely overlapping contrast maps, as shown in Fig. 7b.

To illustrate some possible uses for data fusion and integration and to validate derivatives data, we performed two analysis, one on each dataset. The first one follows the methodology proposed in^27^ to estimate sources localisation using a joint EEG and fMRI sparse model and extend the analysis to XP1 population. The second shows the methodology to learn a fMRI informed EEG filter aiming at improving NF sessions using EEG only, as proposed in^17^: we will synthetically illustrate the methodology in a data integration perspective and show representative results.

### A data fusion approach for joint EEG-fMRI source estimation (XP1)

To estimate source location on the XP1 dataset, we applied the symmetric bi-modal method proposed by^27^ to the motor localizer (motorloc) runs. The proposed approach uses a sparse regularisation for the sources and combines the two modalities EEG and fMRI to improve the spatio-temporal resolution of the source estimation. The problem writes as follow:

5 $$\widehat{{\bf{S}}}{=\arg }_{{\rm{s}}}\min \left\{{\boldsymbol{\alpha }}{\left\Vert {\bf{GS}}-{\bf{E}}\right\Vert }_{2}^{2}+(1-{\boldsymbol{\alpha }}){\left\Vert {\bf{SH}}-{\bf{B}}\right\Vert }_{2}^{2}+{\boldsymbol{\lambda }}{\left\Vert {\bf{S}}\right\Vert }_{1}\right\}$$

**Fig. 8:**
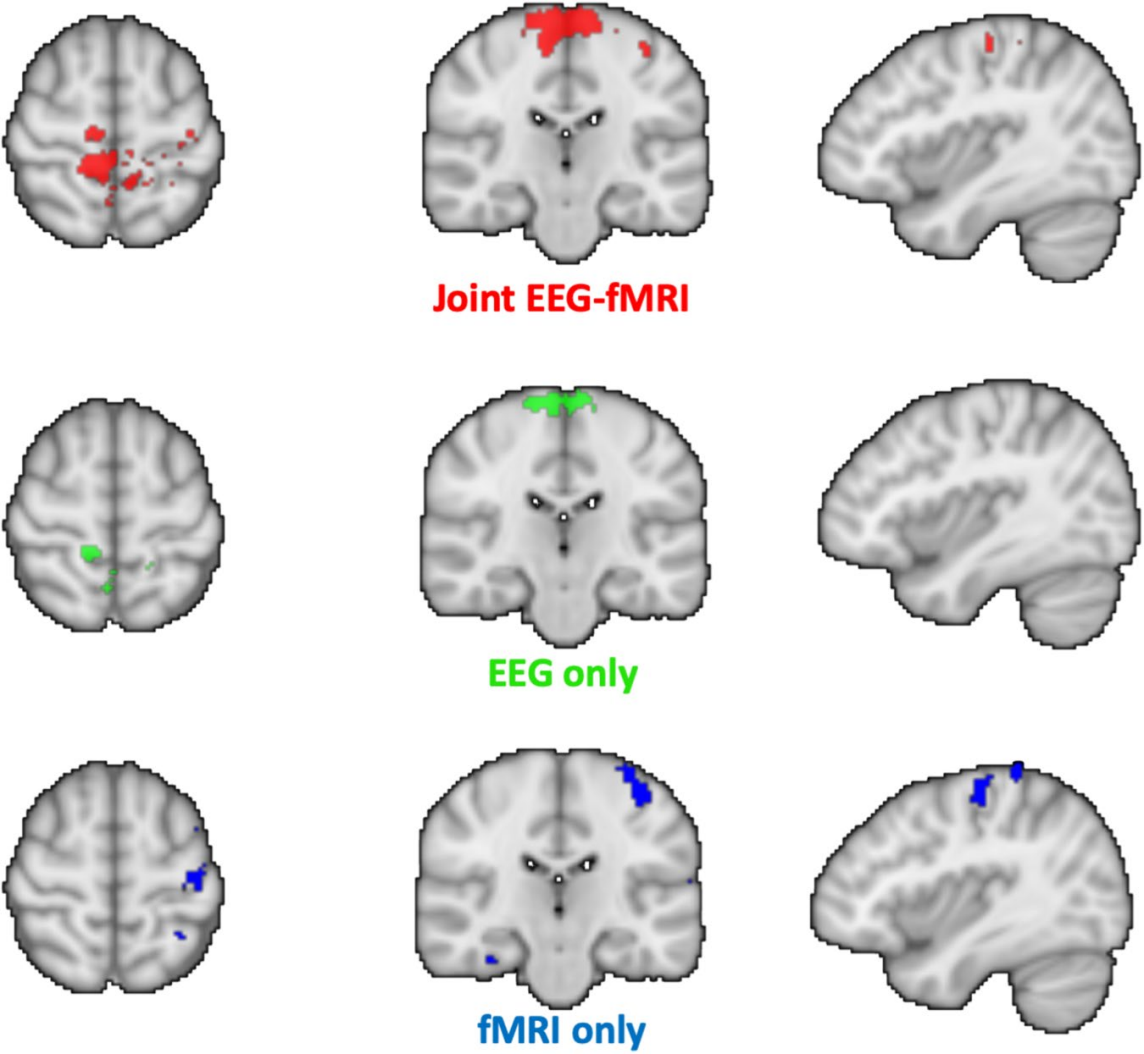
Joint EEG-fMRI source estimation average results. Source locations during motor execution (average across 8 subjects of XP1) estimated using the joint EEG-fMRI sparse model (red), EEG data only (α = 1, green) or fMRI data only (α = 0, blue).

With **S** the estimated sources, **G** the leadfield matrix, **E** the observed EEG signals, **B** the observed BOLD signal and **H** a matrix linking EEG time samples to BOLD time samples using the hemodynamic response function. We chose different values for **α (**1 to consider EEG only, 0.6 the optimal balance between both modalities as found in^27^ and 0 to consider fMRI only), and the optimal sparsity parameter λ = 2. We applied the model on all subjects having performed a motorloc run. Figure 8 shows the average results of source estimation from the joint EEG-fMRI model (**=**0.6), from the EEG only model (**=**1) and from fMRI only model (**=**0). These results indicate that the combination of both modalities allows a more robust and complete localisation of the sources than when using only one modality, as even if fMRI provides a better spatial resolution, it can detect false positives, such as those outside motor areas.

### A sparse EEG-informed fMRI model to improve EEG NF only (XP2)

**Fig. 9:**
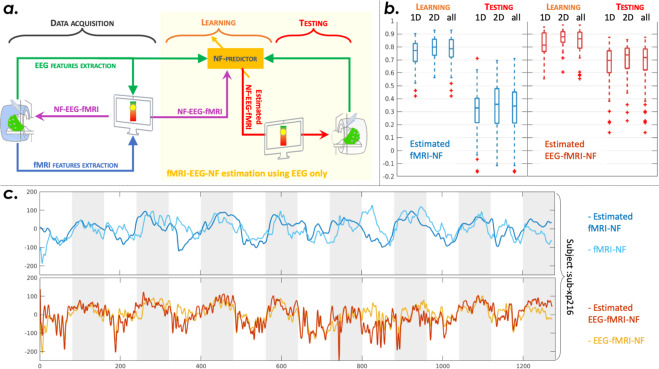
A sparse EEG-informed fMRI model to improve EEG NF only. Panel a.: illustration of the method used to learn fMRI-NF scores from EEG signal. Panel b. shows median and quartiles for correlations between estimated fMRI-NF scores and fMRI-NF scores for all subjects of the dataset XP2, for the learning step and the testing step. Results are also shown for the estimation of the bimodal EEG-fMRI-NF scores from EEG signal only. Panel c.: Example for subject sub-xp216 run 3. The estimated scores were learned on its first NF run, light blue is the ground truth and dark blue the estimated fMRI-NF scores from EEG. Below, the yellow plot shows the bimodal NF score and in red its estimation using EEG only.

We intended to learn fMRI-NF scores from EEG signal as proposed in^17^ in order to determine if fMRI information could be integrated to EEG, on the dataset XP2. The learning and testing process is illustrated in Fig. 9a. The method proposes a model able to learn fMRI-NF scores from bi-modal NF sessions, using EEG signals only. The model learns some parameters of a design matrix composed by different frequency bands between 8 and 30 Hz, and different time delays to better match fMRI delays. A mixed norm is applied (l_1_ on the electrodes dimension and l_21_ on the frequency bands dimension) to the model to regulate the parameters. We excluded from the analysis the two subjects with a bad ERD, as detailed above. For each subject, one of the sessions was used to learn the model, and the two other NF sessions were used to test the model. To validate the efficiency of the prediction, we correlated the estimated fMRI-NF scores with the fMRI-NF scores of the testing sessions, Fig. 9b shows boxplots in blue of such correlation across the dataset, for the learning phase and the testing phase. Information from fMRI could be extracted using EEG signals, as EEG signals could predict fMRI-NF scores with a median correlation of 0.34 with the ground truth. The model is quite well adapted to the data as shown by the learning step scores. Figure 9b also shows the correlation of the estimated EEG-fMRI-NF scores using only EEG-NF scores, with the ground truth that is the EEG-fMRI-NF scores. Figure 9c shows an example of prediction on subject sub-xp216, the estimated NF scores have been learned on its first NF run and tested on its last run.

## Usage Notes

This dataset of simultaneously acquired EEG and fMRI during a NF motor imagery task has potential to shed light on the coupling model underlying the EEG and fMRI signals, to advance methodologies for multimodal data integration and test EEG de-noising methods. The design of the platform and the data collection itself represented a challenge and a veritable research advancement: this constitute a major added value of this dataset but also implies that some technical challenges during data acquisition (especially for the first experiment XP1) led to missing training sessions in some subjects. Another limitation of this dataset is the quality EEG acquired during MRI imaging. As showed in the technical validation section and in previously published work, however, average results are in line with literature thus indicating the overall quality of EEG recordings. New (offline and real-time) EEG artefact correction algorithms may be efficiently tested on these data, as we provided raw and pre-processed EEG data. The efficacy of the proposed algorithms may for instance be assessed looking at quality of ERD patterns associated to the motor imagery (or execution) task. An additional limit of the experimental setup is that hand movements were only monitored online by means of a camera in the MR bore and were not recorded (by measuring the electromyographic signal, for instance). In the MR environment, this type of measure requires an additional amplifier and cable lengths adapted to each individual, thus overburdening an already complex setup. We therefore decided to repeat to participants before each training session to stay still and not to execute the imagined movement and we verified via video monitoring that these instructions were respected.

Even if the definition of the BIDS standard for EEG is still evolving^28^ we tried to adhere to this standard to have an easily and robustly exploitable EEG-MRI BIDS dataset, that can be processed using predefined and standardized pipeline (such as fMRIprep, BIDSHandler and various other BIDS-Apps, https://github.com/BIDS-Apps).

As mentioned in the previous section common and freely available software packages for EEG and MRI data processing were used to analyse data (SPM, Nipype, FieldTrip), except for EEG pre-processing for gradient and BCG artefact correction, for which the Brain Vision Analyser software (Brain Products GmbH, Gilching, Germany) was used. Other scripts used for the Technical Validation in this paper are provided by the authors as specified in the Code Availability session.

## Supplementary information

Supplementary File 1

Supplementary File 2

## Data Availability

A detailed description of the bimodal EEG-fMRI NF platform is given in^20^: the platform software package for real-time analysis and visualization is well documented but not publicly available. Python pipelines for the analysis of structural and functional MRI are available on github (https://github.com/glioi/BIDS_fMRI_analysis_nipype), in form of commented jupyter notebooks. Other scripts used for the technical validation in this paper can be provided by the authors upon request.
