# Supplementary material for: Simultaneous EEG-fMRI during a neurofeedback task, a brain imaging dataset for multimodal data integration
Source: Sci Data. 2020 Jun 10;7:173. doi: 10.1038/s41597-020-0498-3 (PMC7287136; doi:10.1038/s41597-020-0498-3)
Supplement: Supplementary file 1 [file 41597_2020_498_MOESM1_ESM.pdf]

Consensus on the **R**eporting and **E**xperimental **D**esign of clinical and cognitive-behavioural **N**eurofeedback studies (CRED-nf) best practices checklist 2020* (an online tool to complete this checklist is available at rtfin.org/CREDnf).

Domain	Item #	Checklist item	Reported on page #
**Pre-experiment**			
	1a	Pre-register experimental protocol and planned analyses	N.A.
	1b	Justify sample size	N.A.
**Control groups**			
	2a	Employ control group(s) or control condition(s)	p. 4 (XP2)
	2b	When leveraging experimental designs where a double-blind is possible, use a double-blind	N.A.
	2c	Blind those who rate the outcomes, and when possible, the statisticians involved	N.A.
	2d	Examine to what extent participants and experimenters remain blinded	N.A.
	2e	In clinical efficacy studies, employ a standard-of-care intervention group as a benchmark for improvement	N.A.
**Control measures**			
	3a	Collect data on psychosocial factors	N.A.
	3b	Report whether participants were provided with a strategy	p. 3
	3c	Report the strategies participants used	N.A.
	3d	Report methods used for online-data processing and artifact correction	pp. 4-5
	3e	Report condition and group effects for artifacts	N.A.
**Feedback specifications**			
	4a	Report how the online-feature extraction was defined	pp. 4-5
	4b	Report and justify the reinforcement schedule	p.4 + Perronnet et al. 2017, 2018
	4c	Report the feedback modality and content	p. 4
	4d	Collect and report all brain activity variable(s) and/or contrasts used for feedback, as displayed to experimental participants	pp. 4-5 + Perronnet et al. 2017, 2018
	4e	Report the hardware and software used	p.3 + Mano et al. 2017
**Outcome measures**			
Brain	5a	Report neurofeedback regulation success based on the feedback signal	Perronnet et al. 2017, 2018
	5b	Plot within-session and between-session regulation blocks of feedback variable(s), as well as pre-to-post resting baselines or contrasts	Perronnet et al. 2017, 2018
	5c	Statistically compare the experimental condition/group to the control condition(s)/group(s) (not only each group to baseline measures)	Perronnet et al. 2018
Behaviour	6a	Include measures of clinical or behavioural significance, defined a priori, and describe whether they were reached	N.A.

	6b	Run correlational analyses between regulation success and behavioural outcomes	N.A.
**Data storage**			
	7a	Upload all materials, analysis scripts, code, and raw data used for analyses, as well as final values, to an open access data repository, when feasible	p. 14 (Data Citation)

*Darker shaded boxes represent *Essential* checklist items; lightly shaded boxes represent *Encouraged* checklist items. We recommend using this checklist in conjunction with the standardized CRED-nf online tool (rtfin.org/CREDnf) and the CRED-nf article, which explains the motivation behind this checklist and provides details regarding many of the checklist items.
