# Supplementary material for: Simultaneous EEG-fMRI during a neurofeedback task, a brain imaging dataset for multimodal data integration
Source: Sci Data. 2020 Jun 10;7:173. doi: 10.1038/s41597-020-0498-3 (PMC7287136; doi:10.1038/s41597-020-0498-3)
Supplement: Supplementary file 2 [file 41597_2020_498_MOESM2_ESM.pdf]

Comments to CRED-nf list

Control groups

Item 2a: Being these exploratory studies whose aim was to assess the feasibility of bimodal EEG-fMRI-NF (XP1) and how the how the visual NF metaphor would affect NF performance. (XP2), a control group (non-neurofeedback or placebo) was not included in the design. The aim of experiment XP2 was to compare 1D and 2D visual NF, therefore for the 2D group the control condition was the 1D metaphor, in a between-group design.

Control measures

Item 3b: *Patients were instructed to perform a kinaesthetic motor imagery of the right hand and to find their own strategy to control and bring the ball to the target.*

Item 3d: *Real-time correction of EEG artefact was performed using the Brain Vision Recview software. An average artefact subtraction approach was used for gradient artefact correction, with four artefact templates. EEG data were then down-sampled to 200 Hz and low-pass filtered at 50 Hz (48 dB slope) for further processing. BCG artefact correction was then performed using a moving template matching method with the following parameters: pulse period 800ms, correlation threshold 0.7 and amplitude ratio between the period examined and the pulse model ranging from 0.6 td 1.2. The moving pulse template was built averaging the 10 previous detected pulsed. Real-time fMRI pre-processing (slice-time and motion correction) was performed directly by the NF control unit using a custom Matlab script based on SPM 8 (FIL, Wellcome Trust Centre for Neuroimaging, UCL, London, UK). More details, according to the COBIDAS-inspired template, are reported in Table 1.*

Feedback specifications

Item 4a: We reported Online NF features calculation formulas for XP1 and XP2 in pp 4-5.

Item 4b: We reported how feedback was provided and when it was updated in pp. 4-5. More information can be also found in Perronnet et al. 2018 and Perronnet et al. 2017.

During NF runs the screen displayed a white ball moving in the vertical (condition eegNF, EEG laterality depicted the ball ordinate), or horizontal (condition fmriNF, BOLD laterality depicted the ball abscissa) or both dimensions (condition eegfmriNF) and a square representing the target.

Item 4e: Information about hardware and software architecture are provided in p 3 and a very detailed description of the platform for bimodal EEG-fMRI NF in Mano et al 2017.

Outcome measures:

Since this is a dataset descriptor manuscript detailing the structure of the open-access datasets,we did not report in detail outcomes of the NF training protocols as for a clinical or cognitive study. However details about NF scores and activation outcomes can be found in the two related publications: Perronnet et al. 2017 and Perronnet et al. 2018.
